# Uveitis output in high-impact clinical ophthalmology journals: a bibliometric analysis

**DOI:** 10.1186/s12348-025-00490-w

**Published:** 2025-03-25

**Authors:** Baotram V. Nguyen, Priyanka Bhatnagar, Daniel C. Lee, Meghan K. Berkenstock

**Affiliations:** 1 Drexel University College of Medicine, Philadelphia, PA USA; 2 George Washington School of Medicine and Health Sciences, Washington, DC USA; 3 Division of Ocular Immunology, Wilmer Eye Institute, Johns Hopkins School of Medicine, 600 N. Wolfe St., Maumenee Building Third Floor, Baltimore, MD 21087 USA

**Keywords:** Uveitis, Uveitis specialists, Ocular immunology, Research output, Impact factor

## Abstract

**Background:**

Despite uveitis subspecialty workforce shortages, uveitis specialists remain engaged in research. This study examines the relationship between the proportions of uveitis-focused articles in high-impact ophthalmology journals and fellowship-trained uveitis specialists on their editorial boards.

**Methods:**

A bibliometric analysis was conducted on articles published from 2014 to 2023 in the five highest-impact ophthalmology journals: *Ophthalmology*, *JAMA Ophthalmology*, *British Journal of Ophthalmology* (BJO), *American Journal of Ophthalmology* (AJO), and *Investigative Ophthalmology and Visual Science* (IOVS). Editorial board members with uveitis or ocular immunology fellowships were identified from public domain sources. Articles were screened using uveitis MeSH terms. Data analysis was performed using STATA to assess the relationship between the proportions of uveitis-focused articles and uveitis-trained editors.

**Results:**

From 2014 to 2023, 3.57% (575/16,093) of articles published in the five journals were uveitis-focused. The proportion of uveitis-focused articles ranged from 1.74% in *IOVS* to 5.89% in *AJO*. On average, fellowship-trained uveitis specialists comprised 5.28% of editorial board members annually. There were positive correlations between the proportions of uveitis-focused articles and uveitis-trained editors annually (*r* = 0.6799, *p* < 0.00005) and over the 10-year period (*r* = 0.2675, *p* < 0.00005). No significant correlation was observed within individual journals.

**Conclusions:**

Uveitis research remains underrepresented in high-impact ophthalmology journals despite research productivity in the field. While a positive correlation between uveitis-trained editors and uveitis-focused articles was found across all journals, this trend did not hold within individual journals. Enhancing uveitis research visibility in high-impact journals is essential to advancing clinical knowledge, improving patient outcomes, and inspiring ophthalmologists to enter this underserved subspecialty.

**Supplementary Information:**

The online version contains supplementary material available at 10.1186/s12348-025-00490-w.

## Introduction

One of the ophthalmology subspecialties experiencing workforce shortage is uveitis with only 210 fellowship-trained physicians in the United States as of December 2023 [1]. In recent years, vacant uveitis fellowship positions have remained available and unfilled for several cited reasons including the perception of challenging diagnostic problems, limited number of uveitis faculty mentors during residency, and higher hours spent in direct patient care [2]. Despite the heavy clinical workload, uveitis specialists remain highly involved in research. They are more likely to offer a research project to residents and rate “opportunities for research” as more important in career decisions than other ophthalmologists [2]. This trend in research productivity continues even after residency and fellowship, with a recent study showing that uveitis specialists have the highest mean number of publications per author compared to other ophthalmic subspecialties [3].

Despite this high research productivity, it is unclear how likely uveitis publications are to be in high-impact factor journals compared to research in other subspecialties. There are conflicting data on the impact of uveitis-focused publications with authors ranging on both extremes of the mean h-index compared to other ophthalmology subspecialties [3, 4]. One source looking at seven of the top ophthalmology journals found the percentage of publications focused on uveitis was 4.7% [5]. Furthermore, multiple studies assessing the breakdown of publications in high-impact journals by ophthalmology subspecialty omit uveitis as a subspecialty, suggesting that uveitis is not yet universally accepted as its own field of study within ophthalmology [5–7].

Since research productivity is often considered in promotions and faculty appointments at academic medical institutions, it is important to investigate the number of uveitis publications in high-impact journals. This study assesses the percentage of publications in the five highest-ranked impact factor ophthalmology journals focusing on uveitis-related topics in the last 10 years and investigates the relationship between the percentages of uveitis publications and uveitis specialists on each journal’s editorial and advisory boards.

## Materials and methods

### Study identification

The five highest-impact general ophthalmology journals were selected by utilizing data from the Journal Citation Reports 2023 [8]: *Ophthalmology*, *JAMA Ophthalmology*, *British Journal of Ophthalmology* (BJO), *American Journal of Ophthalmology* (AJO), and *Investigative Ophthalmology and Visual Science* (IOVS). A bibliometric analysis was conducted on all original research articles, reviews, and manuscripts published in each database over the past 10 years (2014–2023). This process involved navigating to the “Sources” tab, entering “Ophthalmology” into the Subject Area search bar, and selecting the relevant journals from the search results. From each journal’s homepage, the option to “View all documents” was chosen. The search results were then filtered by publication year (2014–2023) and restricted to research articles and review articles for inclusion in the analysis [9].

Drexel University College of Medicine Internal Review Board deemed this study exempt since all data used was public and constituted non-human subjects research.

### Data collection

The official webpages of the five journals were used to identify the names of the editorial and advisory board members of each journal for each issue (accessed in September 2024). For journals that did not list the editorial board in their issues, the editorial board webpage archived on the Wayback Machine [10] was used to identify board members. Additionally, information about the participating members of each journal’s editorial board was extracted from public domain sources such as faculty profiles. Their length of involvement with their respective journal and whether they are uveitis specialists were recorded. Editors who completed uveitis or ocular immunology fellowships were classified as uveitis specialists. In journals that did not list editors in every issue, the number of uveitis specialists and the number of board members were assumed as average of the preceding and succeeding issues.

**Fig. 1:**
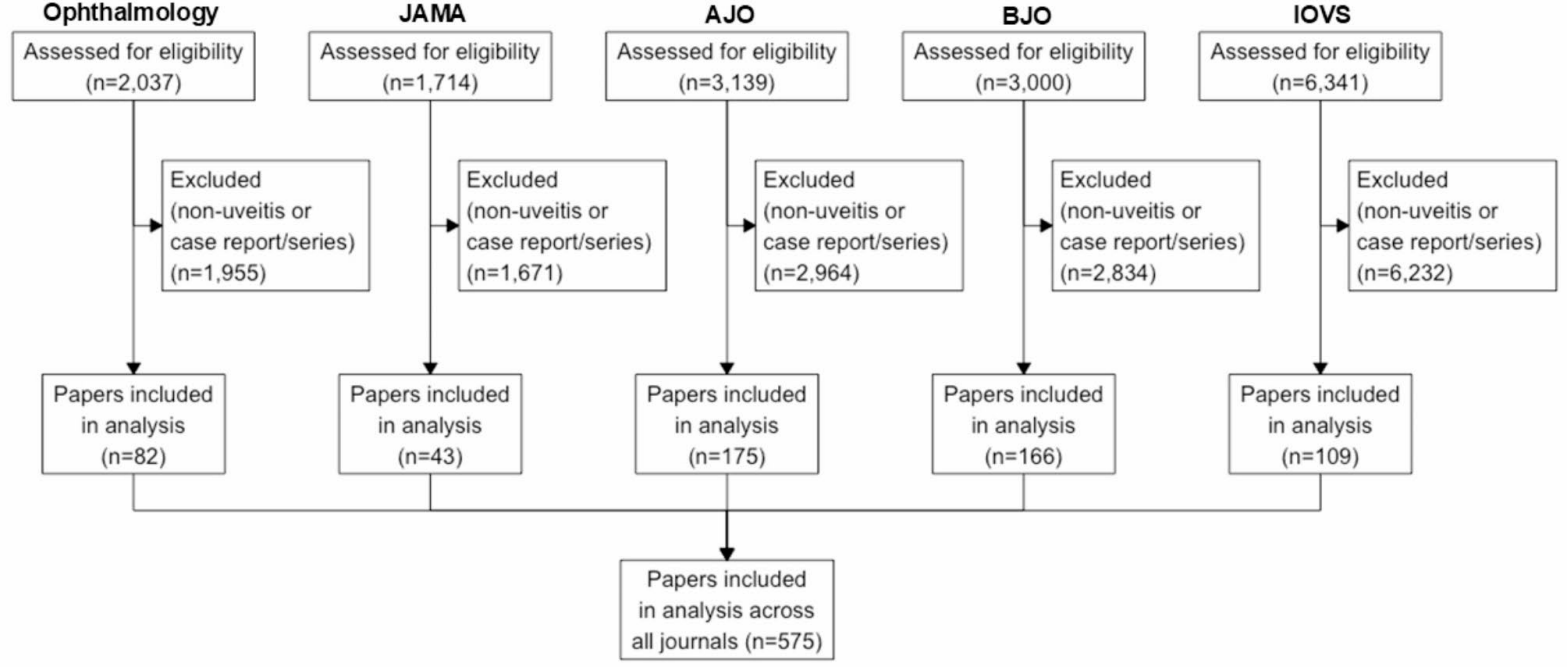
CONSORT diagram showing article selection. Eligibility defined by published between 2014–2023 and document type restricted to “articles” and “reviews” only Abbreviations: JAMA: Journal of the American Medical Association Ophthalmology; BJO: British Journal of Ophthalmology; AJO: American Journal of Ophthalmology; IOVS: Investigative Ophthalmology and Visual Science

The following data were collected for every article in each monthly journal issue within the study period: title, volume, issue, document type, abstract, and index keywords. The document types were restricted to research articles and review articles, thereby excluding case reports, case series, commentaries, response to the editor, and editorials from the initial dataset. Publications were then organized by volume (month) and screened manually for uveitis-related content using all Medical Subject Headings (MeSH) terms associated with uveitis (Appendix 1). Articles that did not include relevant MeSH terms or focused on populations without ocular inflammation were excluded. Three authors (BN, PB, DL) independently reviewed the extracted abstracts to identify eligible articles. When an abstract was unavailable, the full text was reviewed. In cases of disagreement, the principal investigator (MKB) made the final inclusion decision. Figure 1 outlines the article selection process.

### Data analysis

A descriptive analysis was performed on the data of articles and editors from each of the five journals. The primary outcome was the percentage of uveitis-focused articles overall and secondary outcomes included the percentage of uveitis-focused articles per journal per year as well as the percentage of uveitis specialist editors per journal per year. The relationship between the percentage of uveitis-focused articles and the percentage of uveitis specialist editors for each journal was analyzed with Pearson’s correlation coefficient. Statistical significance was determined by *p*-value ≤ 0.05. Data analyses were conducted with STATA (Version 18.0; Stata Corp, College Station, TX). Figures were designed with Excel for Windows (Version 2409 Build 16.0.18025.20030; Microsoft Corp, Redmond, Wash.)

## Results

Overall, 3.57% (N=575) of the 16,093 articles published from 2014 to 2023 across the five selected journals were uveitis-focused (Table 1). The *AJO* had the highest proportion of uveitis articles at 5.89% (175/2973), while *IOVS* had the lowest proportion of uveitis articles at 1.74% (109/6275). The average annual proportion of uveitis articles published was 3.61% (± 0.85), ranging from 2.24 to 5.56% over the study period. No significant trends were observed in the annual proportion of uveitis articles across the decade. Table 1 presents the descriptive analysis of the articles for each journal.

**Table 1:** Proportion of uveitis-focused articles by journal

Journal	Number of uveitis-focused articles	Number of total articles	Proportion of uveitis-focused articles (%)
*Ophthalmology*	82	2037	4.025528
*JAMA Ophthalmology*	43	1725	2.492754
*British Journal of Ophthalmology*	166	3083	5.384366
*American Journal of Ophthalmology*	175	2973	5.88631
*Investigative Ophthalmology and Visual Science*	109	6275	1.737052
All Journals	575	16,093	3.572982

Overall, the proportion of uveitis specialist editors was 5.29% (1704/32,179). The *BJO* had the highest proportion of uveitis editors at 9.26% (391/4224), while *IOVS* had the lowest proportion of uveitis editors at 1.60% (194/12,102). The average annual proportion of uveitis editors was 5.28% (± 0.47), ranging from 4.65 to 5.92% over the study period. No significant trends were observed in the overall annual proportion of uveitis specialist editors over the 10-year span (*r* = 0.0313, *p* = 0.9316). There were also no significant trends in the annual proportion of uveitis editors for *JAMA Ophthalmology* (*r* = -0.478, *p* = 0.1623) or *AJO* (*r* = -0.3489, *p* = 0.3231). The annual proportion of uveitis specialist editors over time increased in *Ophthalmology* (*r* = 0.8787, *p* = 0.0008) and *BJO* (*r* = 0.7414, *p* = 0.0141), but decreased in *IOVS* (*r* = -0.8229, *p* = 0.0035). Table 2 presents the descriptive analysis of the editors for each journal.

**Table 2:** Proportion of uveitis specialist editors by journal

Journal	Number of uveitis specialist editors	Number of total editors	Proportion of uveitis specialist editors (%)
*Ophthalmology*	409	4682	8.735583
*JAMA Ophthalmology*	120	3795	3.162055
*British Journal of Ophthalmology*	391	4224	9.256629
*American Journal of Ophthalmology*	590	7376	7.998915
*Investigative Ophthalmology and Visual Science*	194	12,102	1.603041
All Journals	1704	32,179	5.295379

A positive correlation was identified between the proportion of uveitis articles and the proportion of uveitis specialist editors both annually (*r* = 0.6799, *p* < 0.00005) and over the 10-year period (*r* = 0.2675, *p* < 0.00005) when considering all five selected journals together. However, no significant correlation was observed between the proportion of uveitis articles and editors per year or across the entire 10-year period within each individual journal. Table 3 reports the correlation analysis for each journal.

**Table 3:** Correlation analysis between proportion of uveitis-focused articles and proportion of uveitis specialist editors by journal

Journal	Pearson’s correlation coefficient for proportions by issue	*p* value	Pearson’s correlation coefficient for proportions by year	*p* value
*Ophthalmology*	0.1729	0.0589	0.4283	0.2169
*JAMA Ophthalmology*	0.1594	0.082	0.5304	0.1148
*British Journal of Ophthalmology*	-0.1478	0.1072	0.3073	0.3878
*American Journal of Ophthalmology*	0.0153	0.8679	-0.1704	0.6378
*Investigative Ophthalmology and Visual Science*	0.0847	0.3577	0.2516	0.4831
All journals	0.2675	< 0.00005	0.6799	< 0.00005

## Discussion

Our study evaluated the representation of the field of uveitis in five high-impact ophthalmology journals over a 10-year period, revealing that only 3.57% of published original articles and reviews focused on uveitis. Additionally, uveitis-trained specialists made up an average of 5.28% of editorial board members annually. The positive correlation observed between the proportion of uveitis articles and the proportion of uveitis-trained editors—both annually (*r* = 0.6799, *p* < 0.00005) and over the 10-year period (*r* = 0.2675, *p* < 0.00005)—suggests that editorial board composition may exert an influence on the visibility of uveitis in high-impact ophthalmology literature. The absence of this correlation within individual journals and the generally low representation of uveitis-trained editors could be impacted by sample size. However, several additional factors must be considered when interpreting these findings.

One critical consideration is the relative proportion of uveitis specialists in the ophthalmology community. In the United States, uveitis specialists constitute roughly 1.2% of the total ophthalmology workforce [1, 11]. Despite this, uveitis specialists demonstrate strong research productivity, leading in metrics such as mean number of papers, mean h-index, and a mean annual increase in h-index [3], highlighting the impact of research generated within this field. Our findings suggest that this productivity is not adequately reflected in high-impact ophthalmology literature, indicating a visibility gap. This underrepresentation may have broader implications, as high-impact ophthalmology journals serve as primary resources for ophthalmologists worldwide, providing updates on disease pathophysiology and treatment advances. Limited visibility of uveitis research in these outlets may slow the dissemination of new findings and advancements in care. Additionally, given that publication in high-impact journals is closely tied to academic career progression, restricted exposure may dissuade potential trainees from pursuing careers in this already underserved subspecialty [12].

Several factors may contribute to this limited representation. Editorial and peer-review bias is one consideration, as highly specialized uveitis papers may be perceived as too niche or complex, reducing their acceptance in general ophthalmology journals. Additionally, the interdisciplinary nature of uveitis–spanning rheumatology, infectious disease, and immunology–may prompt specialists to publish in subspecialty journals outside of ophthalmology, further diminishing uveitis representation in clinical ophthalmology literature. Lastly, the lower proportion of uveitis articles in high-impact literature may also reflect the lower proportion of active academic uveitis specialists. Nonetheless, the restricted visibility reinforces the perception of uveitis as a niche and less accessible field, limiting exposure to general ophthalmologists. Enhancing the presence of uveitis research in high-impact general ophthalmology journals could improve awareness, ultimately facilitating better diagnosis and management of these complex conditions. This is particularly critical given the shortage of uveitis specialists in the United States, where only 63.3% of the population resides within an hour’s drive of a uveitis specialist [13]. Limited access to specialized care, especially in rural areas, places a greater responsibility on general ophthalmologists to manage uveitis cases. Expanding the representation of uveitis research in widely-read ophthalmology journals would help bridge this gap by equipping general ophthalmologists with the knowledge needed to navigate these challenging cases.

This study offers a unique, decade-long overview of uveitis publishing trends in high-impact ophthalmology journals and contributes valuable insights into the representation of this subspecialty. However, certain limitations must be acknowledged. Our analysis is restricted to five high-impact journals, which may not fully capture broader trends in uveitis publishing. Furthermore, without access to data on rejected manuscripts, it is difficult to fully assess the role of editorial bias. Data on the proportion of uveitis researchers submitting to high-impact journals relative to the total number of authors was unavailable, raising the possibility that uveitis researchers preferentially submit to uveitis-focused journals. If this is the case, the limited visibility of uveitis research in high-impact ophthalmology journals may be more reflective of submission patterns rather than editorial bias. Future studies should explore uveitis publication trends in interdisciplinary journals and investigate rejection rates to gain a more comprehensive understanding of its representation in ophthalmology literature.

In summary, our findings indicate that uveitis research is underrepresented in high-impact ophthalmology journals despite strong research productivity in the field. Although a positive correlation exists between the presence of uveitis-trained specialists on editorial boards and the volume of uveitis-focused publications in the aggregate data, this relationship was not observed within individual journals. Enhancing the visibility of uveitis research in prominent ophthalmology journals is crucial for advancing clinical knowledge, improving patient care, and motivating the next generation of ophthalmologists to pursue uveitis as a subspecialty.

## Electronic supplementary material

Below is the link to the electronic supplementary material.

Appendix 1: List of MeSH terms and topics used to categorize included articles as uveitisfocused

## Data Availability

The data used and/or analyzed during the study are available on the official webpages of Ophthalmology [www.aaojournal.org], JAMA Ophthalmology [www.jamanetwork.com/journals/jamaophthalmology], British Journal of Ophthalmology [www.bjo.bmj.com], American Journal of Ophthalmology [www.ajo.com], and Investigative Ophthalmology and Visual Science [www.iovs.arvojournals.org].

## Abbreviations

BJO: British Journal of Ophthalmology
AJO: American Journal of Ophthalmology
IOVS: Investigative Ophthalmology and Visual Science
