# Supplementary material for: Uveitis output in high-impact clinical ophthalmology journals: a bibliometric analysis
Source: J Ophthalmic Inflamm Infect. 2025 Mar 25;15:31. doi: 10.1186/s12348-025-00490-w (PMC11937460; doi:10.1186/s12348-025-00490-w)
Supplement: Supplementary file 1 — Appendix 1: List of MeSH terms and topics used to categorize included articles as uveitisfocused [file 12348_2025_490_MOESM1_ESM.pdf]

Appendix 1. List of MeSH terms and topics used to categorize included articles as uveitis-focused

Anterior Uveitis
Behcet Syndrome
- Adamantiades-Behcet Disease
- Behcet Disease
- Behcet Triple Symptom Complex
- Old Silk Route Disease
- Triple-Symptom Complex

Birdshot Chorioretinopathy
- Birdshot Chorioretinitis
- Birdshot Retinochoroiditis
- Birdshot Retinochoroidopathy

Chorioretinitis
Choroiditis
Infectious uveitides
- Aspergillus
- Bartonella
- Candida
- Chikungunya virus
- Coccidioidomycosis
- Cryptococcus
- Cysticercosis
- Cytomegalovirus (CMV)
- Dengue
- Ebola
- Histoplasma
- HSV
- Leprosy
- Leptospirosis
- Lyme disease
- Onchocerciasis
- Pneumocystis jirovecii
- Syphilis
- Toxocara
- Toxoplasmosis
- Tropheryma whippelii
- Tuberculosis
- Varicella-zoster virus (VZV)
- West Nile virus
- Zika virus

Iridocyclitis
- Cyclitis
- Heterochromic Cyclitis

Iritis
Multifocal Choroiditis
Panophthalmitis
Panuveitis
Pars Planitis

Posterior Uveitis
Retinitis
Scleritis
Sclerokeratitis
Suppurative Uveitis
Sympathetic Ophthalmia
Uveitis
Uveitis related to autoimmune conditions

- Ankylosing Spondylitis
- Drug-induced
- Inflammatory Bowel Disease (Crohn's Disease, Ulcerative Colitis)
- Juvenile idiopathic arthritis
- Multiple sclerosis
- Polyarthritis nodosa
- Psoriatic Arthritis
- Reactive Arthritis (Reiter’s syndrome)
- Relapsing polychondritis
- Rheumatoid arthritis (RA)
- Sarcoidosis
- Systemic lupus erythematosus (SLE)
- Tubulointerstitial nephritis and uveitis (TINU) syndrome
- Wegener’s granulomatosis (granulomatosis with polyangiitis)

Uveomeningoencephalitic Syndrome

- Uveomeningoencephalitis
- VKH Syndrome
- Vogt-Koyanagi-Harada Disease
- Vogt-Koyanagi-Harada Syndrome

Vitritis
White Dot Syndromes

- Acute Idiopathic Blind Spot Enlargement Syndrome
- Acute Macular Neuroretinopathy
- Acute Posterior Multifocal Placoid Pigment Epitheliopathy
- APMPPE
- Diffuse Subretinal Fibrosis Uveitis
- MEWDS
- Multiple Evanescent White Dot Syndrome
- Punctate Inner Choroidopathy
- Serpiginous Choroiditis
- Serpiginous Choroidopathy
